# Mitochondrial brain proteome acetylation levels and behavioural responsiveness to amphetamine are altered in mice lacking Sirt3

**DOI:** 10.3389/fphys.2022.948387

**Published:** 2022-09-06

**Authors:** Elena Sidorova-Darmos, Merrick S. Fallah, Richard Logan, Cheng Yu Lin, James H. Eubanks

**Affiliations:** ^1^ Division of Experimental and Translational Neuroscience, Krembil Research Institute, University Health Network, Toronto, Canada; ^2^ Department of Physiology, University of Toronto, Toronto, Canada; ^3^ Department of Pharmacology and Toxicology, University of Toronto, Toronto, Canada; ^4^ Department of Surgery (Neurosurgery), University of Toronto, Toronto, Canada; ^5^ Institute of Medical Science, University of Toronto, Toronto, Canada

**Keywords:** Sirtuin, Sirt3, mitochondria, acetylation, amphetamine, brain

## Abstract

Post-translational modification of mitochondrial proteins represents one mechanism by which the functional activity of mitochondria can be regulated. In the brain, these modifications can influence the functional properties of different neural circuitries. Given that the sirtuin family member Sirt3 represents the primary protein deacetylase enzyme in mitochondria, we tested whether brain mitochondrial proteome acetylation would increase in male or female mice lacking Sirt3. Our results confirm that whole brain mitochondrial proteome acetylation levels are indeed elevated in both sexes of Sirt3-KO mice relative to controls. Consistently, we found the mitochondria of mouse embryonic fibroblast (MEF) cells derived from Sirt3-KO mice were smaller in size, and fewer in number than in wild-type MEFs, and that mitochondrial free calcium levels were elevated within the mitochondria of these cells. As protein acetylation can influence mitochondrial function, and changes in mitochondrial function have been linked to alterations in neural circuit function regulating motor activity and anxiety-like behavior, we tested whether Sirt3-deficient mice would display sensitized responsiveness to the stimulant amphetamine. Both male and female Sirt*3*-KO mice displayed hyper-locomotion and attenuated anxiety-like behavior in response to a dose of amphetamine that was insufficient to promote any behavioural responses in wild-type mice. Collectively, these results confirm that Sirt3 regulates mitochondrial proteome acetylation levels in brain tissue, and that the absence of Sirt3 increases the sensitivity of neural systems to amphetamine-induced behavioural responses.

## 1 Introduction

Converging evidence suggests that altered mitochondrial function may play a role in the pathophysiology of neurological and neuropsychiatric disorders and their associated behavioural disturbances (Johri and Beal, 2012; Müller et al., 2015; Marin and Saneto, 2016). While a number of factors influence mitochondrial functionality, it is now recognized that post-translational modifications (PTM) of the mitochondrial proteome influence mitochondrial homeostasis (Stram and Payne, 2016). One common mitochondrial protein PTM is acetylation, with over 65% of proteins within the mitochondrial proteome being targets for acetylation. In fact, more than 2,000 different acetyl-lysine sites have been mapped in the mitochondrial proteome, with several proteins harbouring multiple acetylation sites (Hebert et al., 2013). Acetylation of lysine residues can occur enzymatically (Freiman and Tjian, 2003) or through non-enzymatic mechanisms involving acetyl-CoA (Wagner and Payne, 2013; Baeza et al., 2015). Deacetylation of these targets occurs primarily, if not exclusively, by enzymatic removal of acetyl groups—through which the exposure of the positively charged lysine residue can affect interactions of the target protein with its respective substrates and cofactors (Hebert et al., 2013).

Within the mitochondria, sirtuin family members represent the primary group of deacetylases that maintain the homeostatic balance of this lysine PTM (Baeza et al., 2014; Baeza et al., 2015). In mammals, the family of sirtuin proteins consists of seven members (SIRT1-7), which are orthologs of the yeast Sir2 enzyme that has been implicated as a longevity-enhancing factor (Gottlieb and Esposito, 1989; Imai, 2001; Dali-Youcef et al., 2007). Of the seven sirtuins, only Sirt3, Sirt4 and Sirt5 predominantly or exclusively reside within the mitochondria (Onyango et al., 2002; Ahuja et al., 2007; Nakamura et al., 2008). While Sirt4 and Sirt5 possesses some deacetylase activity, Sirt3 functions as the primary mitochondrial deacetylase (Lombard et al., 2007), and has been proposed to represent a key regulatory point through which overall mitochondrial function can be dynamically controlled (Bell and Guarente, 2011; Guarente, 2011; Bause and Haigis, 2013; Dai et al., 2014; Salvatori et al., 2017; Xie et al., 2020). As such, alterations in Sirt3 function could play a role in neurological and neuropsychiatric conditions in which altered mitochondrial function and/or homeostatic balance play contributing roles.

The majority of current knowledge regarding Sirt3 functions stems largely from investigations performed in peripheral tissues and non-neuronal cells (Lombard et al., 2007; Hirschey et al., 2011; Fernandez-Marcos et al., 2012; Zheng et al., 2019). Although Sirt3 is widely expressed in the periphery, it is also prominently expressed in different neural cell types and in different brain regions (Sidorova-Darmos et al., 2014; Braidy et al., 2015). Given the high-energy demands of neurons and their dependence on oxidative phosphorylation for ATP production (Sidorova-Darmos et al., 2018), it is likely that Sirt3 also plays a key role in maintaining aspects of neuronal function. However, the specific effects conveyed by Sirt3 to mitochondrial systems and proteome regulation in the brain remains largely unexamined. In this study, we tested whether mitochondrial proteome acetylation patterns would differ in brain tissue isolated from male and female Sirt3-KO mice. Additionally, although previous studies have assessed behavioural consequences due to Sirt3 deficiency (Lombard et al., 2007; Liu et al., 2015), there are no data ascertaining whether the absence of Sirt3 might predispose mice to behavioural responses following neural challenges such as the administration of a low dose of a stimulant. The rationale for this stems from reports indicating the absence of Sirt3 alters mitochondrial efficiency in a way that allows for increased genesis of reactive oxygen species (ROS), which when chronically elevated is known to activate the PI3-kinase/Akt pathway in different cell systems (Sundaresan et al., 2009; Koundouros and Poulogiannis, 2018). Intriguingly, enhanced PI3-kinase/Akt signalling in brain has been linked to behavioural hyper-responsiveness to the administration of psycho-stimulants such as amphetamine (AMPH) (Mines and Jope, 2012; Valjent et al., 2006). In this study, we tested whether male or female Sirt3-KO mice would display any altered behavioural responsiveness to AMPH.

## 2 Results

### 2.1 Mitochondrial mass, number, and Ca^2+^ levels are altered in Sirt3-KO mouse embryonic fibroblasts

**FIGURE 1:**
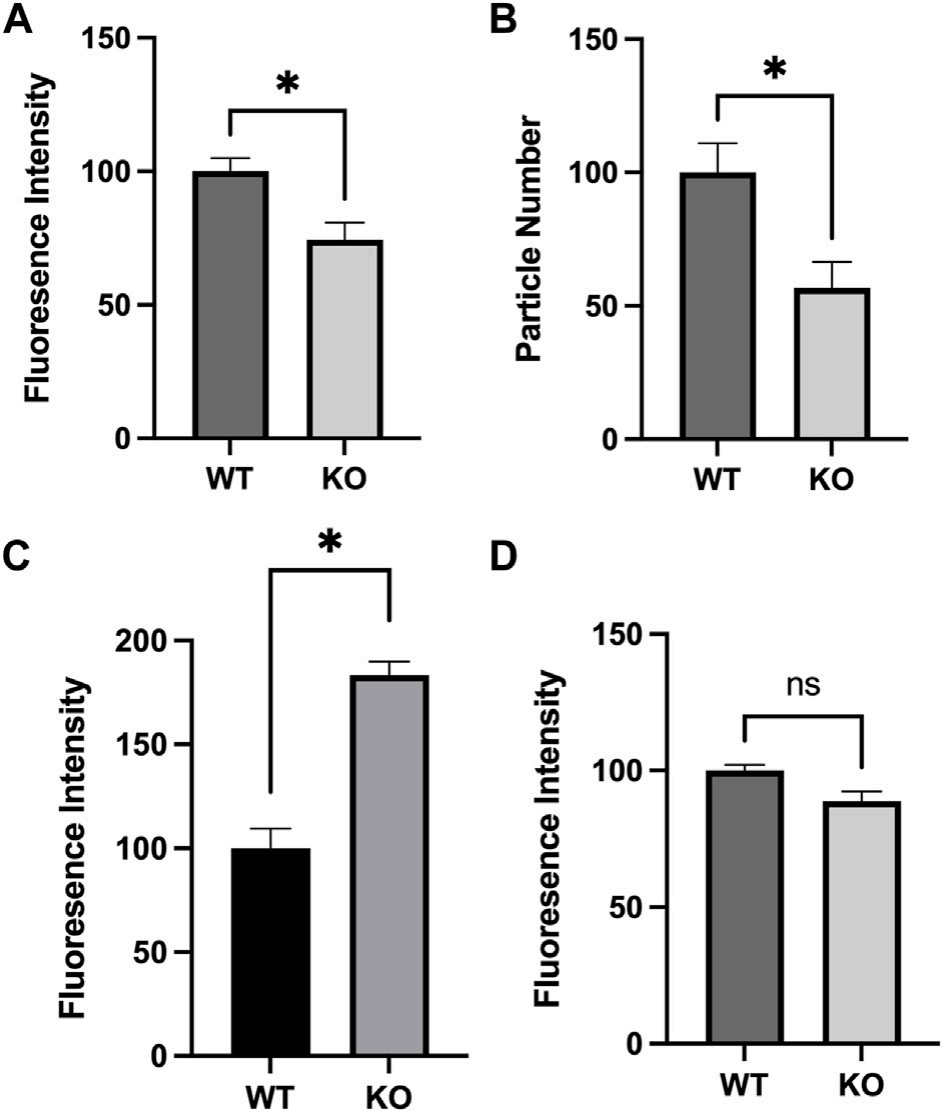
Altered mitochondrial characteristics in Sirt3-KO mouse embryonic fibroblasts. Histograms show the mean and SEM of **(A)** mitochondrial mass, **(B)** count, **(C)** Ca^2+^ concentration, and **(D)** membrane potential measured using flow cytometry and confocal microscopy. MEFS generated from Sirt3-KO mice show significant decreases in mitochondrial size and number, increased mitochondrial Ca^2+^ concentration. No difference in mitochondrial membrane potential was detected. Asterisks denote statistical significance (*p* < 0.05) between the indicated groups (*n* = 4 independent cultures) (Student *t*-tests).

Mitochondria are highly dynamic organelles that can respond to cellular stresses through changes in overall number, mass, mitochondrial membrane potential, and Ca^2+^ levels (Ferree and Shirihai, 2012; Brillo et al., 2021), and the activity of Sirt3 has been previously linked to each of these alterations (Brown et al., 2013; Cheng et al., 2016; Lee et al., 2016; Ren et al., 2017; Castillo et al., 2019). Using MEF lines derived from Sirt3-KO or WT mice as a model system, we expanded on these observations. We first used flow cytometry to compare the average mitochondrial mass within cells labeled with MTGreen. A significant decrease in MTGreen staining intensity was observed in the Sirt3-KO MEFS. As MTGreen binds to mitochondrial proteins independent of membrane potential, this result indicates overall mitochondrial mass is decreased in Sirt3-KO MEFs (Figure 1A). Confocal imaging of MEFs loaded with Rhodamine-123 and unbiased counting was used to compare mitochondrial numbers between cell types. Consistent with the observed decrease in mitochondrial mass, these comparisons revealed diminished mitochondrial numbers per cell in Sirt3-KO MEFs relative to WT (Figure 1B). We then tested whether free mitochondrial Ca^2+^ levels would be altered under basal conditions in cells lacking Sirt3. For this, we used the mitochondrial Ca^2+^ indicator dye Rhodamine-2 (Kosmach et al., 2021). These results revealed that free mitochondrial Ca^2+^ levels were significantly increased in Sirt3-KO MEFs, as indicated by the increased staining intensity of Rhodamine-2 (Figure 1C). In these assays, we also tested whether mitochondrial membrane potential (ΔΨm) would differ between the Sirt3-KO and WT MEF cells. Under these basal conditions, no differences in mitochondrial membrane potential were observed between Rhodamine-123 stained Sirt3-KO and WT MEFs using flow cytometry analysis (Figure 1D). The lack of difference in ΔΨm is an important validation, as the mitochondrial accumulation of the Ca^2+^ indicator dye Rhodamine-2 is influenced by mitochondrial membrane potential (Kosmach et al., 2021). Thus, the increase in Rhodamine-2 signal observed in the Sirt3-KO MEFS reflects increased free mitochondrial Ca^2+^ levels, and not a difference in dye loading efficiency. Taken together, these data confirm that the absence of Sirt3 is sufficient to alter these mitochondrial properties in cells under basal conditions.

### 2.2 Male and female Sirt3-KO mice display mitochondrial proteome hyper-acetylation in the adult brain

**FIGURE 2:**
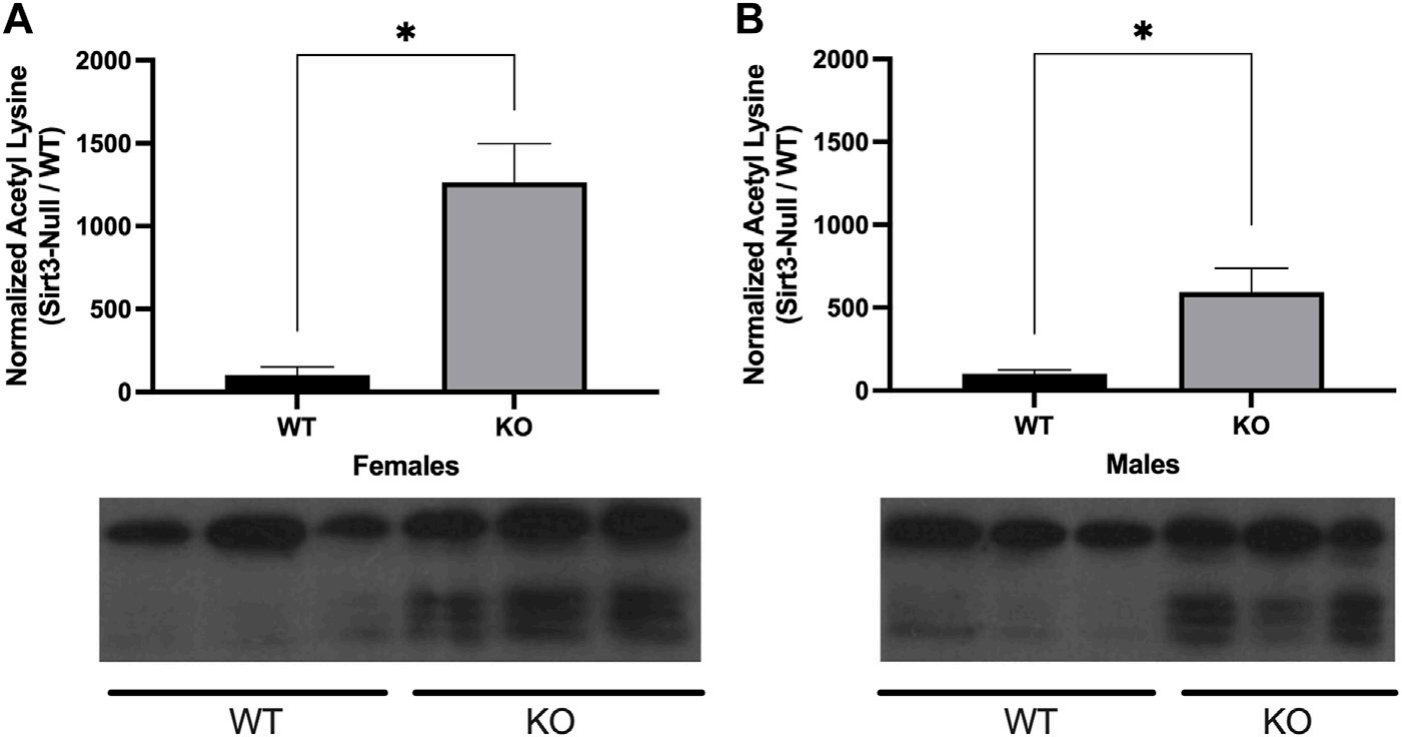
Alterations in lysine acetylation levels of whole brain mitochondrial proteins in Sirt3- KO mice. A representative Western blot showing the immunoreactivity of anti-acetylated lysine. Antibody in mitochondrial fractions isolated from whole brain tissues in male and female WT and Sirt3-KO mice (*n* = 3 independent subjects). **(A)** Female and **(B)** male Sirt3-KO mice have increases of acetylated lysine in proteins isolated from the mitochondrial fraction of whole brain tissues. The histograms show the densitometric mean and SEM for each specific homogenate expressed as a percent ratio over total protein measured using Ponceau staining. Asterisks denote statistical significance (*p* < 0.05) between the indicated groups (*n* = 3 independent subjects) (Student *t*-test).

Previous studies have also shown Sirt3-KO cells and tissues display increased mitochondrial proteome acetylation. These investigations have focused primarily on tissues isolated from male subjects, with hyper-acetylation being observed in mitochondria from liver, and striated muscle tissues, as well as from brain regions such as cortex and hippocampus (Lombard et al., 2007; Hirschey et al., 2011; Fernandez-Marcos et al., 2012; Cheng et al., 2016). To date, however, studies have not examined female Sirt3-KO mice. To address this, mitochondrial fractions from whole-brain tissue of Sirt3-KO and wild-type male and female mice were isolated and assessed for levels of protein acetylation. Female Sirt3-KO mice displayed significantly increased mitochondrial proteome acetylation levels relative to female wild-type mice (Figure 2A). Likewise, and consistent with previous reports (Someya et al., 2010; Weinert et al., 2015; Tyagi et al., 2018), mitochondrial proteome acetylation levels from whole brain were also significantly elevated in male Sirt3-KO mice relative to male wild-type mice (Figure 2B).

### 2.3 Male and female Sirt3-KO mice administered low dose amphetamine display enhanced locomotor behavior in the open field test

**FIGURE 3:**
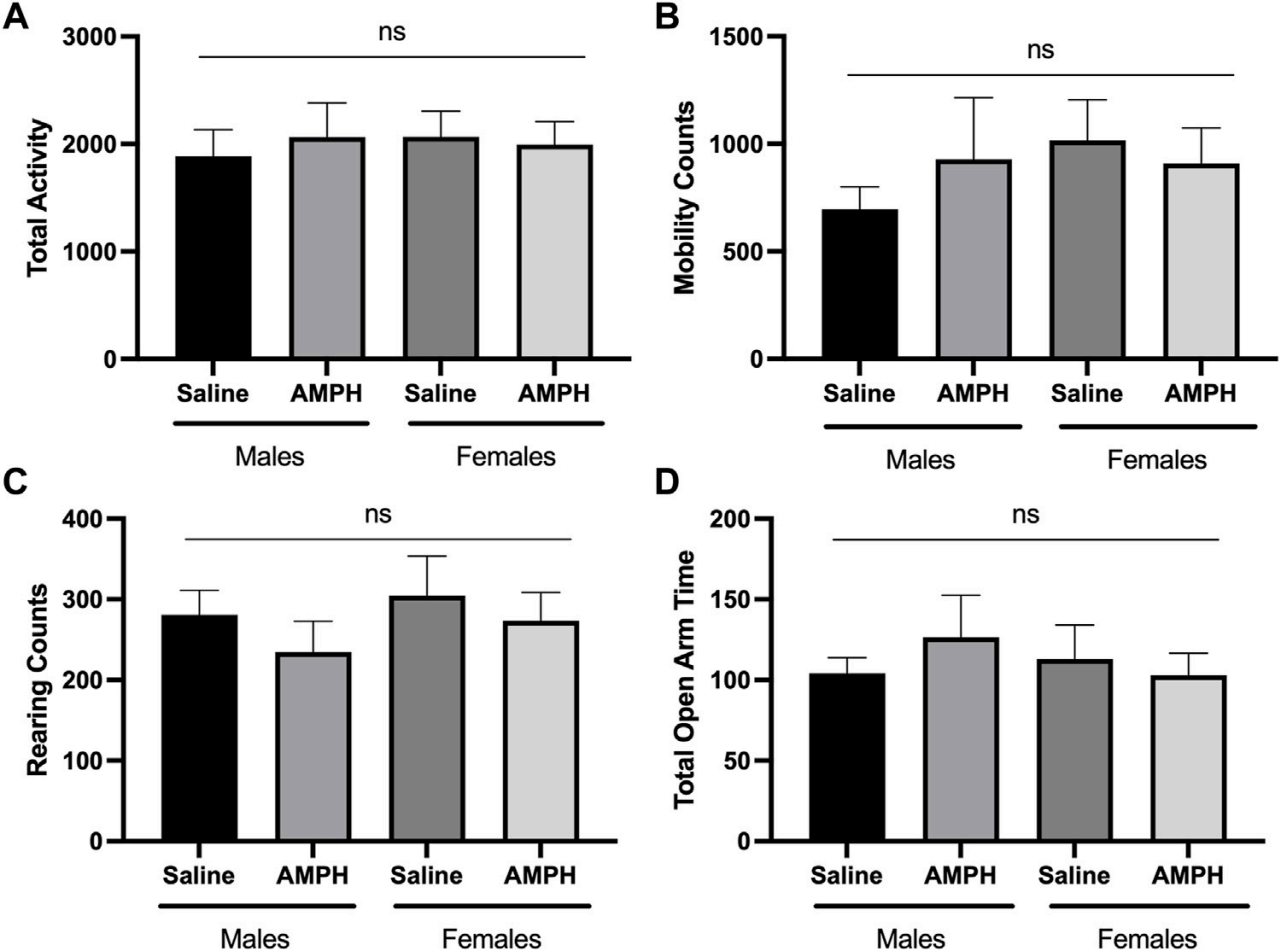
AMPH treatment had no effect on the behavioral response of male and female WT mice in the open-field and elevated plus maze. **(A—C)** Histograms show the mean and SEM of. behavioral performances of WT male and female mice in different open-field assessments treated with either saline or 1 mg/kg AMPH during a 30-min period. Parameters assessed were: **(A)** total activity, **(B)** mobility counts, **(C)** rearing counts. **(D)** Histogram showing the mean and SEM of the elevated plus maze test measuring the total amount of time mice spent in the open areas during a 5-min test period. Data are presented as the behavioral output observed in subjects that received AMPH normalized to those that received saline injection. **(A—D)** There was no statistical difference in performance on any of the measured parameters between saline and AMPH treatments in the WT group, two-way ANOVA, open field (male independent subjects: saline *n* = 8, AMPH *n* = 7; female independent subjects: saline *n* = 8, AMPH *n* = 9), elevated plus maze (male independent subjects: saline *n* = 8, AMPH *n* = 9; female independent subjects: saline *n* = 7, AMPH *n* = 9).

**FIGURE 4:**
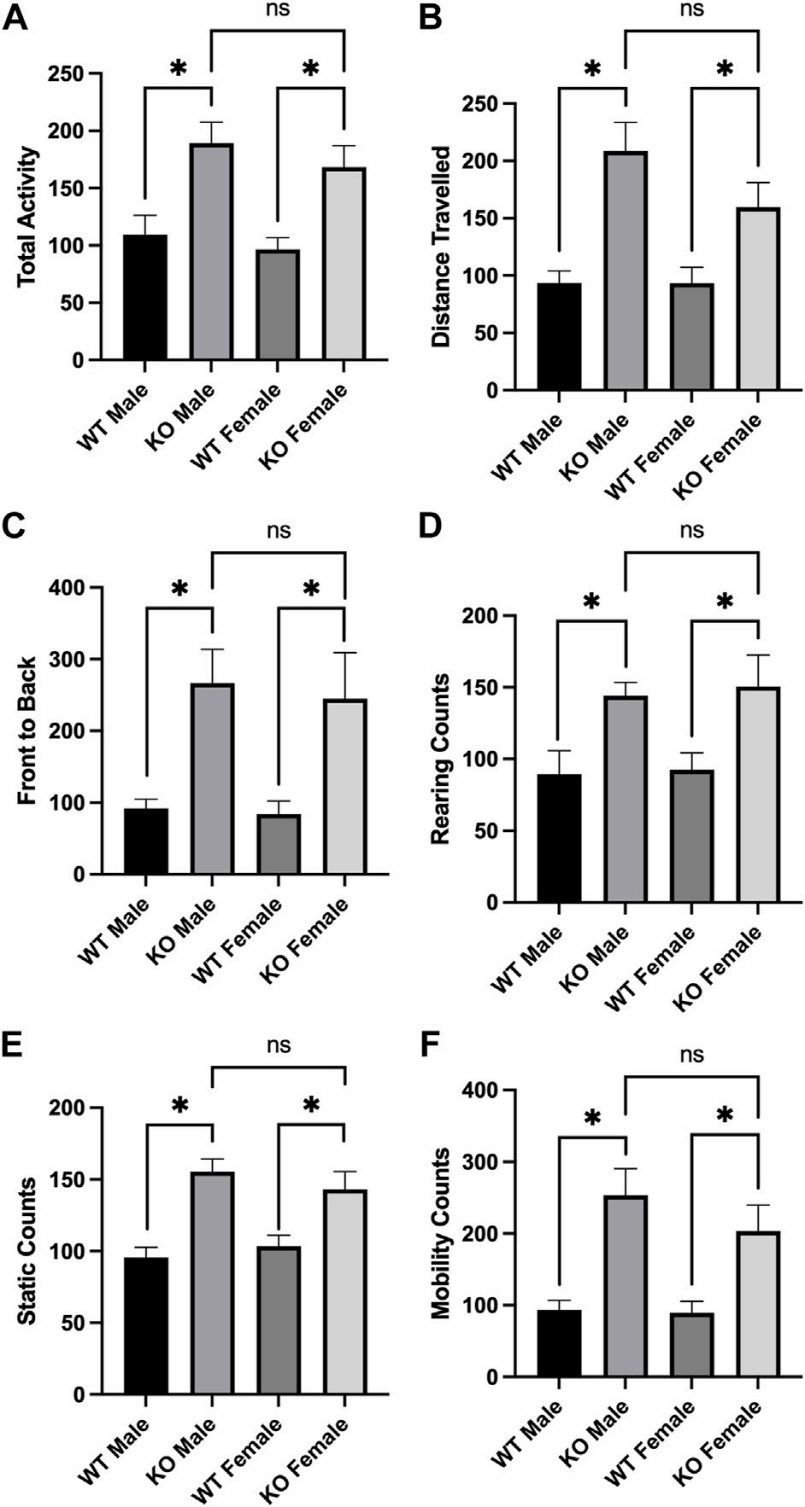
AMPH treatment induced locomotor activity in Sirt3-KO male and female mice in the open field. **(A—F)** Histograms showing the mean and SEM of behavioral performances of WT and Sirt3-KO male and female mice in different open-field assessments treated with either saline or 1 mg/kg AMPH during a 30-min period. Parameters assessed were: **(A)** total activity, **(B)** distance travelled, **(C)** front to back, **(D)** rearing counts, **(E)** static counts and **(F)** mobility counts. Data are presented as the behavioral output observed in subjects that received AMPH normalized to those that received saline injection **(A—F)**. AMPH treatment elevated all assessed behavioral parameters in both male and female Sirt3-KO mice, relative to saline group. Asterisks denote. (*p* < 0.05), two-way ANOVA (male independent subjects: saline *n* = 6, AMPH *n* = 7; female independent subjects: saline *n* = 8, AMPH *n* = 9).

Alterations in locomotor activity have been linked to changes in mitochondrial number, Ca^2+^ levels, and proteome acetylation (Einat et al., 2005; Fukada et al., 2012; Kaska et al., 2017), and elevating Sirt3 expression has also been associated with motor function improvement in a model of Parkinson’s Disease (Gleave et al., 2017). Although Sirt3 deficiency alone is not sufficient to induce alterations in baseline voluntary locomotion (Liu et al., 2015), we questioned whether male and/or female Sirt3-KO mice might display altered sensitivity to a stimulant that induces locomotor activity–especially given the alterations in mitochondrial numbers and free Ca^2+^ levels seen in Sirt3-KO cells (Figure 1C), and the increase in brain mitochondrial acetyl-lysine levels present in Sirt3-KO mice shown in Figure 2. AMPH is a stimulant that can induce locomotor behaviour in mice in a dose-dependent manner (Heal et al., 2013). To test whether Sirt3-KO mice possess heightened sensitivity to AMPH-induced behavioural effects, we first identified a sub-threshold dose of AMPH that was incapable of inducing activity alterations in male and female WT mice. While administration of 3 mg/kg to WT mice induced a robust locomotor response as reported previously (Salahpour et al., 2008), we found that the administration of a 1.0 mg/kg dose of AMPH did not affect locomotor activity in either male or female WT mice (Figure 3). The same 1.0 mg/kg AMPH administration to Sirt3-KO mice, however, resulted in a robust increase of activity (Figure 4). Analysis of open field activity revealed that total activity, total mobility counts, total static counts, rearing counts, total distance covered, and the number of complete front to back cage traversions increased significantly for both male and female Sirt3-KO mice relative to either sex-matched Sirt3-KO mice treated with saline, or to WT mice treated with the same low-dose (1.0 mg/kg) of AMPH (Figure 4).

### 2.4 Male and female Sirt3-KO mice administered low dose amphetamine display decreased anxiety-like behaviour relative to wild-type mice

**FIGURE 5:**
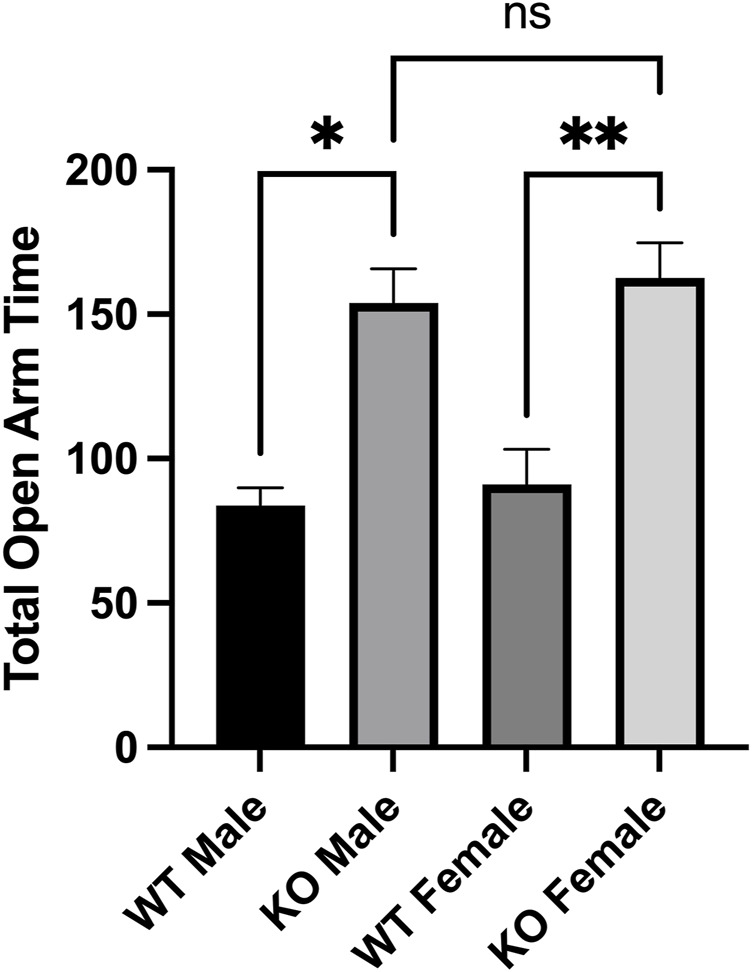
AMPH treatment reduced anxiety-like behaviors in Sirt3-KO male and female mice in the elevated plus maze paradigm. **(A-B)** Histograms show the mean and SEM of the plus maze test measuring the total amount that male and female mice treated with either saline or 1 mg/kg AMPH spent in the open areas of the elevated plus maze during a 5-min test period. Data are presented as the behavioral output observed in subjects that received AMPH normalized to those that received saline injection. **(A-B)** AMPH treated male and female mice spent more time in the open arms than those treated with saline. Asterisks denote (*p* < 0.05), two-way ANOVA (male independent subjects: saline *n* = 8, AMPH *n* = 9; female independent subjects: saline *n* = 7, AMPH *n* = 9).

Previous studies have also demonstrated that changes in acetylation PTM levels have an effect on anxiety-like behaviours (Fukada et al., 2012; Sakharkar et al., 2014). Similar to its effects on locomotor activity, AMPH administration is also associated with changes in anxiety-like behaviour in rodents (Silva et al., 2002). The elevated plus maze (EPM) has been used previously used to assess the effects of AMPH on rodent behaviour (Dawson et al., 1995; Silva et al., 2002). Consistent with the lack of effect on locomotor behaviour, the administration of 1.0 mg/kg AMPH failed to alter the performance of either male or female WT mice on the elevated plus maze (Figure 3). Both male and female Sirt3-KO mice receiving this same dose (1.0 mg/kg) of AMPH, however, were found to spend significantly more time in the open arms of the maze relative to their respective Sirt3-KO vehicle controls (Figure 5). As WT mice receiving higher doses of AMPH (3.0 mg/kg) also display decreased anxiety-like behaviour in the same elevated plus maze task (Silva et al., 2002), these results further indicate the absence of Sirt3 sensitizes mice to AMPH-induced behavioural responses.

## 3 Discussion

Protein acetylation is recognized as a mechanism through which different aspects of cellular function can be regulated (Choudhary et al., 2009; Sotnikov et al., 2014; Drazic et al., 2016). Alterations in normal PTM states of neuronal proteins–such as histones and tubulin—have been identified in several neurological conditions as well as pre-clinical model systems (Magiera et al., 2018; Fallah et al., 2021). Within the brain, increased acetyl-histone levels in the medial prefrontal cortex, amygdala, and cingulate cortex of mice (Whittle et al., 2016; Sah et al., 2019), as well as the basal nucleus of stria terminalis of rats (Ranjan et al., 2017), have been linked to increased locomotor and/or reduced anxiety and fear-like behaviours. Similarly, global loss of tubulin de-tyrosination in the mouse brain is associated with hyper-locomotion, impaired social interaction, and decreased anxiety-like behaviours (Pagnamenta et al., 2019). These results illustrate that when considering mechanisms that could underlie circuit or behavioural consequences, specific modifications to the proteome should also be considered in addition to transcriptome or proteome fingerprints.

The effects of alterations in mitochondrial proteome PTMs on behaviour or brain circuitry functions have been less well investigated to date. Napoli et al., 2017 reported a link between a specific PTM of a mitochondrial protein and an impact on behaviour. This study found that male mice displaying decreased *S*-palmitoylation of mitochondrial-localized dynamin-related protein-1 (Drp1) in cortex and cerebellum displayed hypoactivity, decreased motor coordination, and increased anxiety. Sirt3 represents the predominant deacetylase enzyme present in mitochondria, and the present study shows that its absence in the brain significantly increases mitochondrial proteome acetylation levels in both male and female mice. Given the potential link between altered mitochondrial PTM states and behaviour, we speculated that Sirt3-KO mice might also display a more robust response than WT mice following a stimulus that promotes locomotor and anti-anxiety-like behavioural responses. AMPH is one such stimulant (Wayment et al., 1998; Fleckenstein et al., 2007) whose administration in mice generates dose-dependent increases in voluntary locomotor activity and alters anxiety-like behaviour in the open field ambulation and elevated plus maze tests, respectively (Silva et al., 2002; Salahpour et al., 2008). Both male and female Sirt3-KO mice displayed heightened sensitivity to AMPH-induced behavioural responses in both tasks, and both sexes demonstrated the same magnitude of hyperactivity and diminished anxiety-like behaviour in response to a low dose of AMPH. Importantly, the dose of AMPH employed that promoted clear behavioural responses in both male and female Sirt3-KO mice elicited no response in either male or female wild-type mice. These results confirm that the absence of Sirt3 sensitizes neural circuits within the brain to AMPH-induced behavioural responses and raise the possibility that alterations in Sirt3 activity could similarly affect the sensitivity of these circuits to other psychoactive substances.

The mechanism through which the absence of Sirt3 sensitizes neural systems to AMPH remains unclear. We are unaware of any studies that have examined any direct effects of Sirt3 on presynaptic monoamine transporter expression or function. However, Sirt3 activity has been shown to be requisite for GABAergic synaptic adaptation in response to fasting, and in its absence this GABAergic adaptation does not properly occur (Liu et al., 2019). As GABAergic tone plays a key role in amphetamine-type stimulant use disorders (Jiao et al., 2015), it is possible the absence of Sirt3 predisposes the GABAergic system such that greater behavioural responsiveness emerges following low dose AMPH exposure. Alternatively, previous studies have reported elevations of ROS and increased mitochondrial oxidative stress in Sirt3-KO systems (Sundaresan et al., 2009; Bell and Guarente, 2011; Kim et al., 2011; Bause and Haigis, 2013; Geng et al., 2018). Our results from Sirt3-KO MEFs are consistent with these reported alterations in mitochondrial function. Specifically, decreases in both mitochondrial mass and number, as well as increases in free mitochondrial Ca^2+^ levels, are consistent with increased levels of mitochondrial oxidative stress and ROS (Görlach et al., 2015; Bertero and Maack, 2018). Indeed, increases in oxidative stress has been previously shown in similar Sirt3-KO MEF cell lines (Kim et al., 2010; Geng et al., 2018), and several studies have demonstrated elevation of mitochondrial Ca^2+^ is directly linked to increases in ROS (Görlach et al., 2015; Bertero and Maack, 2018). Such elevations in ROS can have a number of consequences depending on magnitude, but one effect of interest that has been reported in Sirt3-KO systems is the indirect activation of the Ras-PI_3_-kinase-Akt signalling system (Sundaresan et al., 2008). An increase in Akt signalling in the brain would be expected to facilitate increased mTORC1 activity, which intriguingly has previously been linked to the hyper-responsiveness of neural systems to AMPH (Huang et al., 2018). Thus, it is plausible that the observed behavioural sensitization in male and female Sirt3-KO mice to AMPH stems from the enhancement of Akt activity that arises from chronically elevated mitochondrial Ca^2+^ and ROS levels in Sirt3-deficient mitochondria. If correct, then other systems regulated by Akt signalling in the brain may also display sensitization as a consequence of Sirt3 deficiency.

In conclusion, these data add to the knowledge of Sirt3 influence on brain and behaviour. Our results illustrate that both male and female adult Sirt3-KO mice display hyper-acetylation of mitochondrial proteins in brain relative to WT mice, and that the absence of Sirt3 significantly increases the sensitivity of mice to AMPH-induced behavioural responses. These results reveal that the absence of Sirt3 is sufficient to sensitize behavioural responsiveness to stimulants such as AMPH, and raise the question of whether other neural systems may display a similar alteration in sensitivity to context-dependent challenges.

## 4 Materials and methods

### 4.1 Animal use

Animal experiments were conducted in accordance with the guidelines of the Canadian Council of Animal Care, and protocols for animal use were reviewed and approved by the local animal care committee of the University Health Network before commencement of the study. Sirt3-KO mice (cat # 012755; Lombard et al., 2007) and wild-type (WT) C57Bl/6 (cat #000664) mice were purchased from Jackson Laboratories, and breeding colonies were established in house. The original Sirt3-KO mice purchased were on a 129S1/SvImJ background and were backcrossed for at least 8 generations onto a C57Bl/6J background before use. After the backcrossing was done to generate Sirt3 mutant mice on the C57Bl/6J background, Sirt3 heterozygous and WT mice from the same litter were mated, and male and female heterozygous mice from those litters were then crossed to generate the Sirt3-KO and most of the WT mice used for our assays. For some replication studies, WT mice of the appropriate age and sex, and same C57Bl/6J background, were purchased from Jackson Laboratories. No difference was observed in any tested parameter between the in-house bred WT mice and those purchased from Jackson labs. Mice were group housed at 4-5 per cage in a vivarium maintained at 22–23°C with standard 12-h light on/off cycle commencing at 06:00. Food and water were provided *ad libitum*. Experimental studies employed both Sirt3-KO and WT male and female mice that were 5-8 months old. Behavioural assessments were carried out between 08:00 and 13:00 to minimize the effect of circadian rhythms.

### 4.2 Generation of primary murine embryonic fibroblast cultures

Mouse embryonic fibroblast (MEF)-Sirt3-KO cells and MEF-WT cells were isolated from E16 embryos from either Sirt3-KO or WT mice. After isolation, the embryos were transferred to PBS, the heads and internal organs removed, and the remaining material digested with trypsin-EDTA for 30 min at 37°C, 5% CO_2._ The dissociated material was then further triturated to break up cell clumps, and trypsin was inactivated by the addition of FBS. The dissociated cells were plated into a 10-cm plates in DMEM supplemented with 10% FBS, 0.1 mM β-mercaptoethanol, and an antibiotic–antimycotic mixture. The cell culture media was then changed 24 h later. To maintain the healthy proliferation of MEFs, the maximum number of passages did not exceed 3.

### 4.3 Flow cytometry assessments

Sirt3-KO and WT MEFs were plated on 6-well plates at 300,000 cells/well. Each experimental n-number represents experiments conducted on independent cultures that were collected at different times. For each experiment, at least 10,000 events were collected and analyzed for mean fluorescence intensity for positive stained cells. A CytoFLEX LX flow cytometer and Cell Quest program was used to capture and analyze results using dotplot or histogram function.

#### 4.3.1 Mitochondrial mass: MitoTracker green FM

Mitochondrial mass was assessed with the dye MitoTacker Green FM (MTGreen) (Molecular Probes, Cat. # M-7514). After 24 h, cells were incubated with 40 nM MTGreen for 30 min in 5% CO_2_. Positive controls were incubated with 400 nM MTGreen, and negative control cells contained no dye to allow autofluorescence gating. After incubation, cells were collected in PBS containing propidium iodide (PI) (1ug/ml) and analyzed using flow cytometry. PI positive cells were used to gate compromised cells, and the MTGreen signal (419/516 nm) of the remaining viable cells was analyzed using the FL1 channel to obtain the geometric mean fluorescence intensity.

#### 4.3.2 Mitochondrial membrane potential: Rhodamine-123

Basal mitochondrial membrane potential (ΔΨm) was measured using rhodamine-123 (R-302, Invitrogen). After 24 h in culture, cells were incubated for 30 min with 0.5 µM of rhodamine-123 at 37°C in 5% CO_2_. After incubation, the cells were collected in PBS containing PI (1ug/ml) and analyzed using flow cytometry. Two specific controls were employed for this experiment. One control involved treating cells 12 h prior to harvest with the mitochondrial uncoupling agent carbonyl cyanide m-chloro phenyl hydrazone (CCCP) (10 µM) (Sigma, #C-2759), which decreases ΔΨm by facilitating proton movement across the mitochondrial membrane. The second control involved loading cells with a high concentration of rhodamine-123 (20 µM), to ensure the indicator was functioning within the non-quenched range (Perry et al., 2011). Rhodamine-123 signal (505/534 nm) was then collected and analyzed.

#### 4.3.3 Mitochondrial Ca^2+^ levels: Rhodamine-2

Mitochondrial Ca^2+^ levels were measured using mitochondrial-specific probe rhodamine-2 under basal conditions (R-1244, Invitrogen). For basal measurements, the MEFs were incubated with rhodamine-2 (2 mM) for 30 min at 37°C in 5% CO_2_. The cells were then collected in PBS containing PI (1ug/mL) and analyzed by flow cytometry. PI-positive cells were gated out, and viable cells analyzed. Cells not receiving dye were used to gate out autofluorescence. Rhodamine-2 signal (552/581 nm) was collected and analyzed.

### 4.4 Confocal microscopy analysis of mitochondrial number

Sirt3-KO and WT MEFs were plated on 35 mm glass bottomed dishes (WillCo Wells) at a density of 50,000 per dish in MEF culturing medium. MEFs were loaded with 0.5 µM Rhodamine-123 for 30 min, rinsed, and returned to culture medium. Cells were imaged using an inverted scanning confocal microscope (Bio-Rad MRC-600) equipped with an argon-ion laser and BHS filter block. Images were collected using a 60x objective, with a neutral density filter setting of 2, at a zoom scale of 3.0. Mitochondrial morphology from the collected images was analyzed using Image J software.

### 4.5 Mitochondrial fractionation

Mitochondrial purification was conducted as previously described (Chinopoulos et al., 2011), with all steps carried out at 4°C. In short, whole brains from WT and Sirt3-KO male and female littermate mice were individually homogenized in MSEGTA Buffer (225 mM mannitol, 75 mM sucrose, 5 mM HEPES (pH 7.4), 1 mM EGTA, dissolved in water) that was supplemented with 0.2 mg/ml bovine serum albumin (BSA) and centrifuged at ∼500 x g x 5 min. The supernatant was then centrifuged at 14,000 x g x 10 min. The resulting pellet was resuspended in 200 μl of 12% Percoll-MSEGTA solution (100% Percoll-MSEGTA buffer: 225 mM mannitol, 75 mM sucrose, 5 mM HEPES (pH 7.4), 1 mM EGTA, dissolved in 100% Percoll), and this suspension was then layered over 1 ml of 24% Percoll-MSEGTA solution. The prepared density gradient was then centrifuged at 18,000 x g x 15 min. Following centrifugation, 700 μl was aspirated off the top portion of the sample and 1.2 ml of MSEGTA was added and mixed by inversion and centrifuged at 18,000 x g x 5 min. After centrifugation, 1.5 ml of supernatant was aspirated and 1.5 ml of MSEGTA was added and mixed by inversion. The resulting sample was centrifuged at 14,000 x g x 5 min. The pellet was then lysed in lysis buffer [50 mM Tris—pH 8.0, 1% NP40, 150 mM NaCl, 1 mM EDTA, 1mM PMSF, 1 μg/ml Aprotinin, 1 μg/ml Leupeptin, 2 mM Na3VO4, and supplemented with 1 tablet of protease inhibitor cocktail (Roche, Mississauga, Ontario, cat # 11836153001)]. The samples were then centrifuged at 21,000 × g for 10 min. The supernatant was then collected and resulting protein samples were stored at -80°C.

### 4.6 Western blotting

Protein concentration of western blot samples was determined using the Bio-Rad protein assay (Biorad, Mississauga, Ontario, cat # 162-0115). 20 μg of total protein were resolved using western blotting as described in Sidorova-Darmos et al., 2014. Western blots were probed with primary anti-acetyl-lysine antibodies (Rabbit, polyclonal 1:1,000 dilution; Cell Signalling, cat # 9441S) for 24 h at room temperature. Following extensive washing in TBS-T buffer, the blots were incubated with secondary HRP-linked secondary anti-rabbit antibodies (sheep, polyclonal 1:5,000 dilution; GE Healthcare; cat # NA934V). Protein levels were detected using chemiluminescence (GE Healthcare, Amersham ECL Western Blotting Detection Reagents) onto an X-Ray film (Biotex). Protein levels for each product were normalized relative to Ponceau S (Sigma-Aldrich, Oakville, Ontario, cat # 6226-79-5) (Supplementary Figure S4). The optical density of target band was quantified using Image J version 1.816 software (https://imagej.nih.gov/ij/), with background intensity subtracted to give the final densitometric values.

### 4.7 Amphetamine treatment

Amphetamine was obtained in accordance with guidelines established by Health Canada.

*D*-amphetamine sulfate (AMPH) (R&D Systems Europe, cat # 2813) was dissolved in normal saline. AMPH was injected subcutaneously at a volume of 0.01 ml/g. Experimental mice received a single injection of either AMPH (1 mg/kg) or saline vehicle. The injection-test interval was 45-min as this corresponds to the peak activity window observed in the open field following AMPH treatment in a previous study (Yates et al., 2007). Subjects were then assayed in either the open field or elevated plus maze tests.

#### 4.8.1 Open field test

Mice were evaluated for their ambulatory and rearing activity in the open field arena using an automated monitoring system (Linton Instruments, UK) over a 60-min period. The following parameters were tested: total activity, total mobility counts, total static counts, rearing counts, total distance covered, and the number of complete front to back cage traversions.

#### 4.8.2 Elevated plus-maze test

The elevated plus maze (Pellow and File, 1986) apparatus was composed of two open arms (30 cm × 5 cm) illuminated by overhead lights, and two closed arms (30 cm × 5cm) with sidewalls extending 19 cm upwards. Each arm radiated from a central platform (5 × 5 cm), and the apparatus itself was placed at a height of 50 cm above the floor. The test began with a mouse being placed on the central platform, facing one of the two open arms, and was allowed to explore the maze for 5 min while being video recorded. A mouse was scored as entering an arm when each of its four paws were clearly within an arm and assigned as being in the open platform otherwise.

### 4.9 Statistical analysis

GraphPad Prism version 9.00 (GraphPad Software, San Diego CA) was used for all statistical assessments and *p* < 0.05 was set as the level of statistical significance. Student’s *t*-tests and Two-way Analysis of Variance (ANOVA) with post-hoc Tukey tests were conducted where appropriate.

## Data Availability

The original contributions presented in the study are included in the article/supplementary material, further inquiries can be directed to the corresponding author.
