# Supplementary figures and images for: Mitochondrial brain proteome acetylation levels and behavioural responsiveness to amphetamine are altered in mice lacking Sirt3
Source: Front Physiol. 2022 Sep 6;13:948387. doi: 10.3389/fphys.2022.948387 (PMC9489219; doi:10.3389/fphys.2022.948387)

## Slide 1
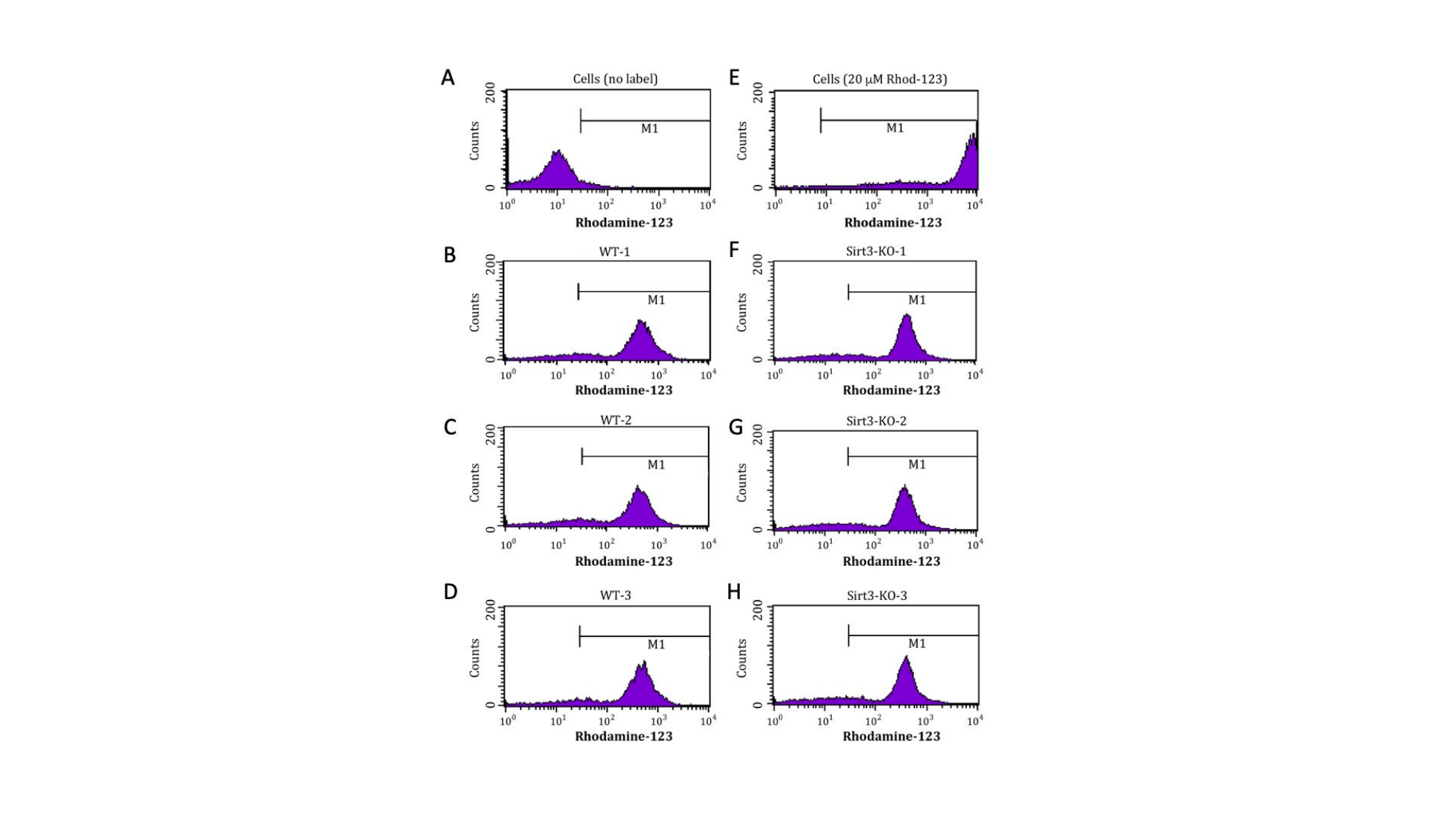

## Slide 2
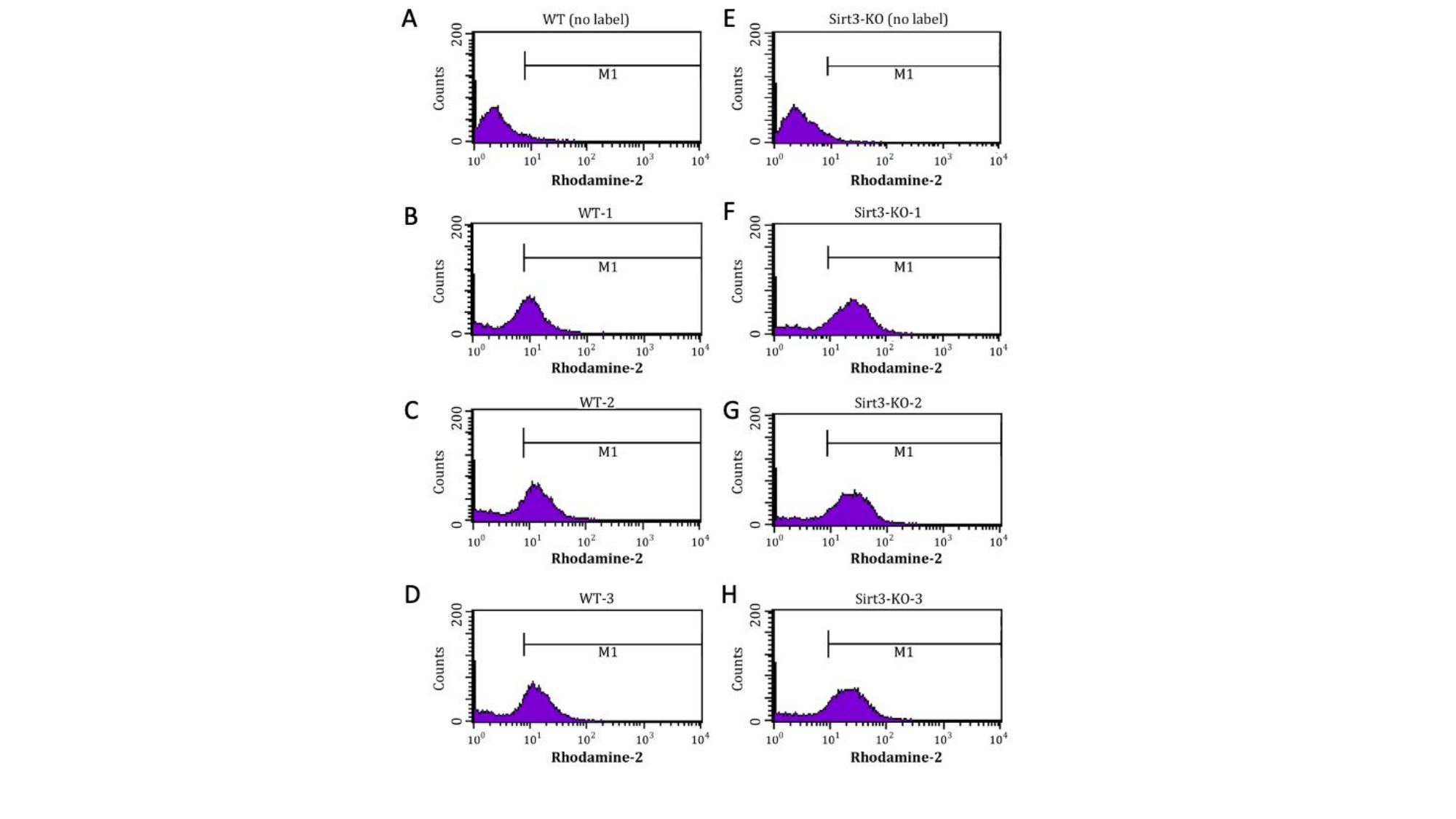

## Slide 3
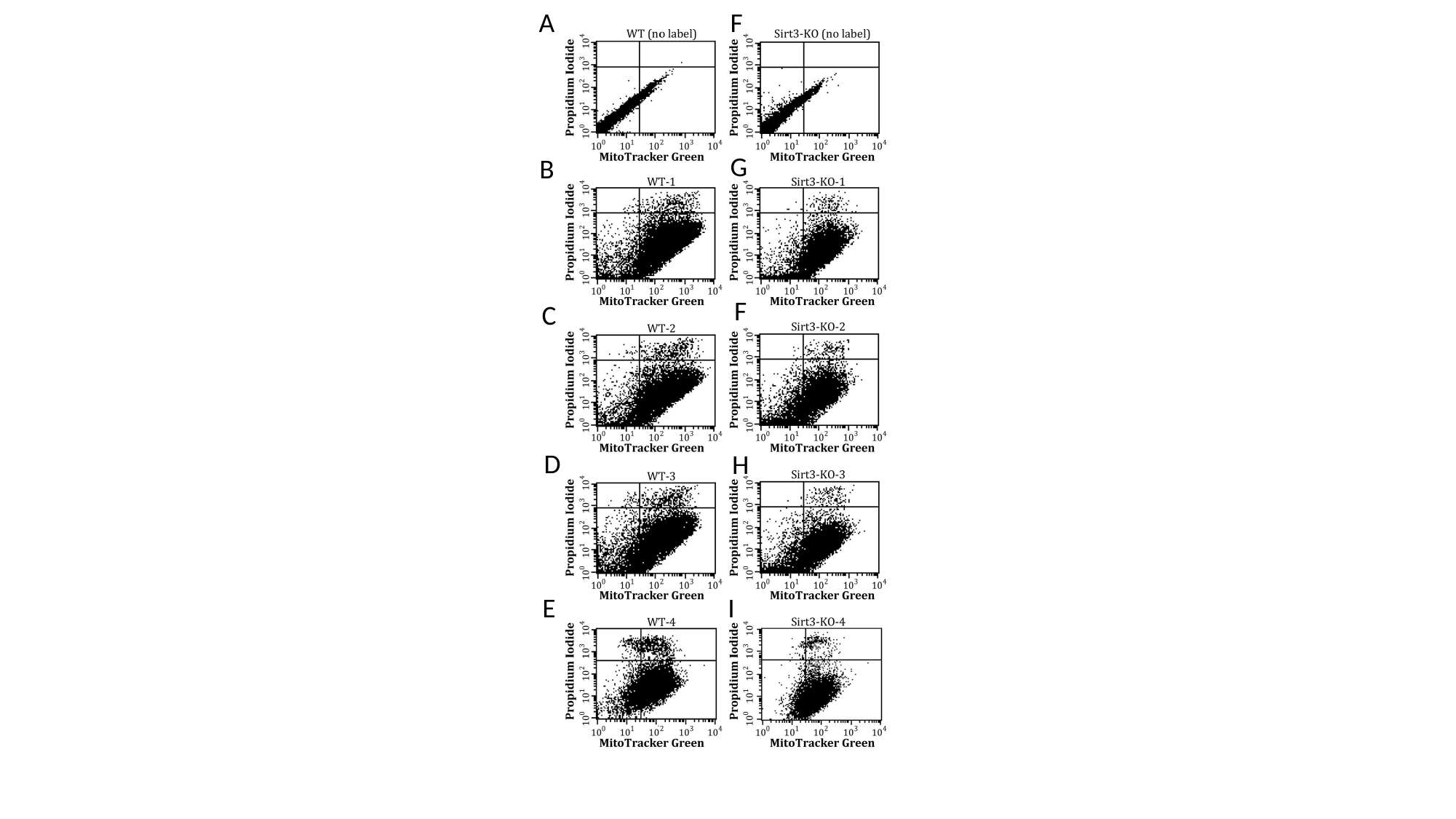

A
F
G
B
F
C
D
H
E
I

## Slide 4
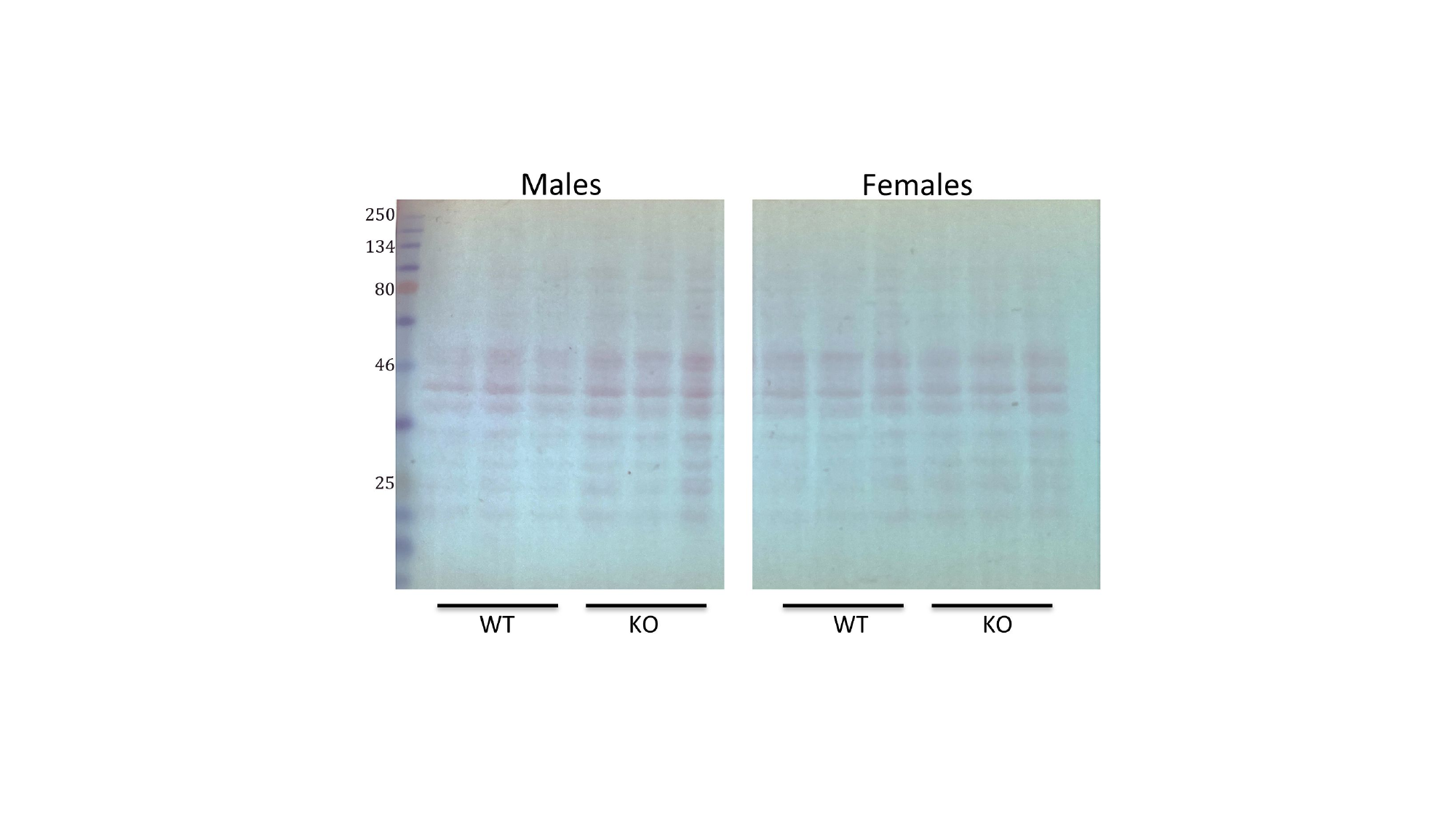

Supplement: Supplementary file 1 [file Presentation1.PPTX]
